# Fish with red fluorescent eyes forage more efficiently under dim, blue-green light conditions

**DOI:** 10.1186/s12898-017-0127-y

**Published:** 2017-04-20

**Authors:** Ulrike Katharina Harant, Nicolaas Karel Michiels

**Affiliations:** 1 Department of Animal Evolutionary Ecology, Institution for Evolution and Ecology, University of Tuebingen, Auf der Morgenstelle 28, 72076 Tuebingen, Germany; 2 Department of Biology, Faculty of Science, University of Tuebingen, Auf der Morgenstelle 28, 72076 Tuebingen, Germany

**Keywords:** Foraging success, Visual ecology, *Tripterygion delaisi*

## Abstract

**Background:**

Natural red fluorescence is particularly conspicuous in the eyes of some small, benthic, predatory fishes. Fluorescence also increases in relative efficiency with increasing depth, which has generated speculation about its possible function as a “light organ” to detect cryptic organisms under bluish light. Here we investigate whether foraging success is improved under ambient conditions that make red fluorescence stand out more, using the triplefin *Tripterygion delaisi* as a model system. We repeatedly presented 10 copepods to individual fish (*n* = 40) kept under a narrow blue-green spectrum and compared their performance with that under a broad spectrum with the same overall brightness. The experiment was repeated for two levels of brightness, a shaded one representing 0.4% of the light present at the surface and a heavily shaded one with about 0.01% of the surface brightness.

**Results:**

Fish were 7% more successful at catching copepods under the narrow, fluorescence-friendly spectrum than under the broad spectrum. However, this effect was significant under the heavily shaded light treatment only.

**Conclusions:**

This outcome corroborates previous predictions that fluorescence may be an adaptation to blue-green, heavily shaded environments, which coincides with the opportunistic biology of this species that lives in the transition zone between exposed and heavily shaded microhabitats.

**Electronic supplementary material:**

The online version of this article (doi:10.1186/s12898-017-0127-y) contains supplementary material, which is available to authorized users.

## Background

Fluorescence is a common form of luminescence that can be found throughout the entire biotic world [1]. The functionality of fluorescence for intra-specific communication has already been studied in a variety of organisms within terrestrial as well as aquatic habitats [2–4]. Especially in aquatic environments, where long wavelengths are quickly absorbed, fluorescence allows organisms to restore long-wavelength color patterns by absorbing the abundant photons in the blue-green spectral range and reemitting some of that energy as light at longer wavelengths. This situation applies to fairy wrasses for example, where it has been shown experimentally that the fluorescence pattern in males plays a role in sexual interactions [5, 6].

Red fluorescence is present in many reef fishes [7, 8]. In small, benthic, predatory fishes, it is often the eyes that fluoresce and they do so more efficiently in deeper water [9, 10]. This depth effect combined with findings that red fluorescence is also phenotypically flexible [11] and becomes more efficient in fish kept in dim environments [12], suggests an optimization to ambient light conditions. Given that several red fluorescent fish can also perceive their own fluorescence [6, 13], we hypothesize that fish with strongly red fluorescent irides may use fluorescence to illuminate and probe their surrounding environment [14]. More specifically, we argue that this form of fluorescence could theoretically be used to induce reflective eyeshine in small prey such as copepods, aiding in their detection. Such active photolocation where prey is illuminated by some kind of private light source has recently been shown in nocturnal flashlight fish [15]. These produce bioluminescent light pulses that might be strong enough to reveal retro-reflection in the eyes of other fish and/or prey nearby. Red fluorescence could be used in a similar way under daylight conditions. This, however, seems more plausible under the heavily shaded, blue-green stenospectral light conditions at depth rather than in shallow, broadly lit euryspectral conditions [9]. We define the euryspectral zone as the depth range close to the surface, with an ambient spectrum that is broader than the visual spectrum of most animals. The stenospectral zone, in contrast, describes the depth range below this, where most of the UV and longer wavelengths have been absorbed by the water column [16, 17], resulting in a spectrum that is narrower than the perception limits of most fish [9, 10]. The transition between the two can be between 5 and 25 m, depending on light conditions and variation in light attenuation by the water column.

Benthic copepods and other micro-crustaceans are a common food source for small fish and their nauplius eyes show strong reflection due to the presence of tapetal cells [18–21]. Own observations and tests (unpubl. data) indicate that copepod eyes reflect incoming light more to the source than elsewhere, similar to a weak retroreflector. Inducing reflective eyeshine in such eyes could therefore be enhanced if the light source (= red fluorescent iris) is close to the pupil, as is also the case for the light organ below the pupil in flashlight fishes [22]. Here, we do not assess whether *Tripterygion delaisi* actually induces and perceives this eyeshine in copepods, but rather examine whether the association between ambient light conditions and foraging behavior is consistent with this hypothesis. More specifically, we test whether fish capture more copepods under bluish light conditions that make fluorescence stand out more compared to broad illumination of the same overall brightness as predicted by our hypothesis. Our model species is the black-faced triplefin *Tripterygion delaisi* (Cadenàt and Blache 1970) [23], a small crypto-benthic fish with strongly red fluorescent irides [12, 24]. Since *T. delaisi* increases the relative efficiency of its fluorescence with decreasing ambient brightness, foraging success was tested under two different brightness levels, mimicking 2° of shading. By doing so, we could assess whether foraging success increases under stenospectral conditions in general, or whether it also requires heavily shaded light conditions.

## Methods

We collected *T. delaisi* while SCUBA diving at Stareso (Station de Recherches Sous Marines et Océanographiques) Calvi, Corsica, France in June 2014 and 2015. After transfer to the aquarium facilities at the University Tübingen, Germany, they were held separately in 40, 15 L tanks which were equipped with a living rock as a comfort stone, in a common water recirculation system (20 °C, salinity 34‰, pH 8.2, 12 h light/dark cycle, fed once per day).

### Tank illumination

Each aquarium was illuminated with a combined set of 8 LEDs in a single housing covered with a Feno Fe s.soft lt 18 diffuser. The LEDs available were: cold white, UV (395–410 nm), royal blue (450–465 nm), blue (465–485 nm), 2× green (520–535 nm), amber (585–595 nm) and red (620–630 nm). Each LED of each housing could be individually controlled by a DMX standalone unit (Feno fc s.dmx 48d) from off (= 0) to maximum (= 100) allowing spectral shape and brightness to be programmed.

### Copepod culture and pilot study on copepod behavior

As a prey model species, we used *Tigriopus californicus* (Baker 1912) a marine harpacticoid copepod that colonizes rock pools from Alaska to Baja California, Mexico [25]. Copepods were cultivated in 1 L tanks (20 °C and 34  ‰ salinity, 12 h light/dark cycle) and fed on a variety of unicellular algae and bacteria. For each of the two experiments, we carried out a pilot experiment in which we tested the preference for the light treatments (stenospectral versus euryspectral) of the copepods. Copepods were inserted into transparent 4 mL cuvettes containing seawater and illuminated each for 2 min with the light treatments used in the main experiment in random order. We then assessed whether the copepods spent significantly more time in the upper or lower half of the cuvette, indicating a preference for a particular light treatment presented. No significant differences were detected (Additional file 1).

### First experiment: spectral treatments under shaded conditions

**Fig. 1:**
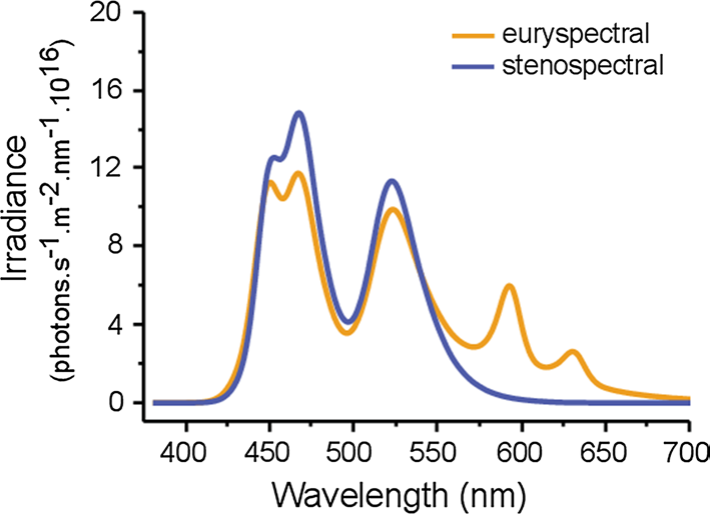
Comparison of spectral shape between euryspectral and stenospectral treatment used in the first (*shaded*) experiment

The experimental room in which fish were kept was divided into two benches with 20 aquaria (total *n* = 40). On 10 October 2014 each bench either received a euryspectral or stenospectral treatment with an identical overall irradiance (total irradiance in photons s^−1^ m^−2^, stenospectral: 2.51 × 10^18^, euryspectral: 2.55 × 10^18^ as in Harant et al. [12]) which represents 3.6% of the total light present just below the water surface on a sunny summer day (Fig. 1). The natural spectrum was measured on the 26 June 2015 at solar noon, close to solar maximum in Corsica, France. These two experimental spectra were designed to mimic the ambient spectra at 5 and 20 m depth, the range in which *T. delaisi* is most abundant. Both benches received a different spectral treatment, alternating every week for 4 weeks. After analyzing the data, we extended the experimental duration for another 2 weeks to confirm the insignificance of the results (6 weeks total).

### Second experiment: same spectra, heavily shaded light conditions

In the second experiment, we used a newly collected set of fish for a test under identical conditions, except that now, brightness was reduced to the lowest level that was manageable to carry out the experiment (about 0.01% of the surface; stenospectral_1_: 7.05 × 10^16^, stenospectral_2_: 7.05 × 10^16^, euryspectral: 7.04 × 10^16^). This involved reducing the light produced by the LEDs electronically, but not below a 6% level, where flickering becomes an issue. Since the red LED was already at a low setting in the euryspectral treatment during the first (shaded) experiment, it could not be lowered more. In order to achieve a low light level we therefore added a cap on top of the light diffuser made of 2 layers of neutral density filter (LEE Filters Nr. 210 0.6 ND) which allowed about 6% of the total light intensity to pass.

**Fig. 2:**
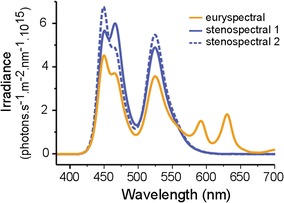
Comparison of spectral shape between euryspectral and stenospectral 1–2 used in the second (*heavily shaded*) experiment

In *T. delaisi*, brightness perception is mainly mediated by the double cones. According to microspectrophotometric measurements, these peak at 516 and 530 nm. Hence, highest sensitivity in this species lies within the green spectral range [26]. The stenospectral treatments containing more green wavelengths compared to the euryspectral treatment could therefore be perceived as being much brighter regardless of the total brightness. To prevent an increase of foraging success due to this perceived brightness effect, we ran two stenospectral treatments which varied by 10% in the amount of green wavelengths (Fig. 2). However, in order to keep the overall brightness identical, the amount of blue light in the second stenospectral treatment was slightly increased (Fig. 2).

Starting from 26 January 2015, each bench received either a euryspectral or stenospectral light condition, which was swapped after each week for 4 weeks. In the stenospectral treatment the two stenospectral alternatives were changed on a daily basis.

### Aquaria experimental setup

The two sides of each aquarium were covered with white non-fluorescing, polypropylene sheets (matt/semi-gloss) sprayed with a fine greyish noise pattern (Hybrid Lack, silver-gray RAL 7001—Additional file 2). This was done to provide a noisy background under the (untested) assumption that it would make it harder for the fish to detect copepods by achromatic contrast alone.

### Recording setup

When testing two individual fish in a pair of aquaria, we fitted a GoPro Hero 3+ above each tank, providing a full view of the bottom of the aquarium where the fish move about. In the second experiment, the light was too dim for good recordings, requiring infrared illumination (Versiton SAL-30 IR Illuminator 77 LEDS 30 M (100′) 12VDC 1.5A, peak at 844 nm). For this, the cameras were fitted with a dedicated IR lens (Vision Dimension: 2.97 mm Megapixel M12 × 0.5). In order to minimize the unlikely possibility of sensitivity to strong IR, the IR sources were positioned at ground level in the room, oriented upwards, but not into the tanks. The diffuse reflection from the ceiling and walls was just bright enough to obtain good recordings (Additional file 3).

### Fish habituation and testing

Prior to the start of the experiment, fish were familiarized with the pipette that was used to provide copepods: UKH frequently inserted the pipette into the aquaria several times a day during the pre-experimental weeks. In the beginning, the pipettes contained defrosted *Mysis* shrimp (Aki Frost GmbH) which were released into the aquarium (3–4 *Mysis* per insertion). As soon as no flight response was observed anymore, seawater was delivered instead of food. This procedure made sure that the appearance of a pipette triggered positive anticipation without guaranteeing food.

After starting the light treatments, fish were allowed to adapt for 1 week without any other change in maintenance conditions. On the following day (Monday), 5 randomly chosen aquaria pairs (*n* = 20 individuals) in each treatment were tested by injecting 10 copepods in 1 mL seawater 2 cm from the front glass using an automatic pipette. The next day (Tuesday) the remaining 10 aquarium pairs (n = 20 individuals) followed. Upon completing the testing procedure, the spectral treatments were changed to the opposite treatment. The same procedure was then repeated again (Monday/Tuesday) under the second spectral treatment the week after. Each pair of aquaria was tested twice per testing day with one fish first receiving copepods whereas the other only received water from the copepod culture as a control to check for feeding strikes due to odor only. In the second run of the day, the role of positive treatment and control was reversed in each aquarium pair. Between the first and second run in a single aquarium pair, another aquarium pair in the opposite treatment (and bench) was tested to induce a delay between the two successive trials in a single aquarium pair. This 10 min delay allowed fish to go back to their normal routine.

Pre-experimental trials showed that fish usually stop searching for copepods after about 5 min. In addition to that, copepods started to hide within the comfort stone (living rock) after 5–6 min after injection. Hence, if fish were not able to catch all copepods within the first run, the chance of detecting leftover copepods still left in the tank during the control treatment in the second run was small (but see "Results" Section).

### Work flow, copepod preparation and video analysis

To enhance video quality, the water inflow of the aquarium was turned off 10 min prior to testing. The copepods were gently taken up by the pipette and released into the aquarium. After insertion of the pipette, videos were recorded for the following 5 min. To prevent observer bias, recorded videos were randomized and transformed to grayscale before analysis. The inserted copepods were too small to be seen on the video, leaving the observer also blind to the copepod treatment and its control. *T. delaisi* shows a saltatory searching behavior [27, 28] which is characterized by approaching prey with small hopping movements, interrupted by scanning of the substrate and a sudden feeding strike once prey is identified. In a pilot study we found that the number of feeding strikes closely approximated the number of live copepods added to the tank, and never exceeding that number. It confirmed that there are no feeding strikes without copepods, and most or all feeding strikes also resulted in successful prey capture. Only rarely, fish needed two strikes in rapid succession for the same item. Such cases were counted as one strike. Overall, the results show that feeding strikes are a reliable variable for measuring foraging success in *T. delaisi* (Additional file 4). In the main experiments, we used Etholog 2.2.5 [29] to record time (s) for each feeding strike since start of the recording as well as total *n* feeding strikes.

### Iris fluorescence of *T. delaisi*

**Fig. 3:**
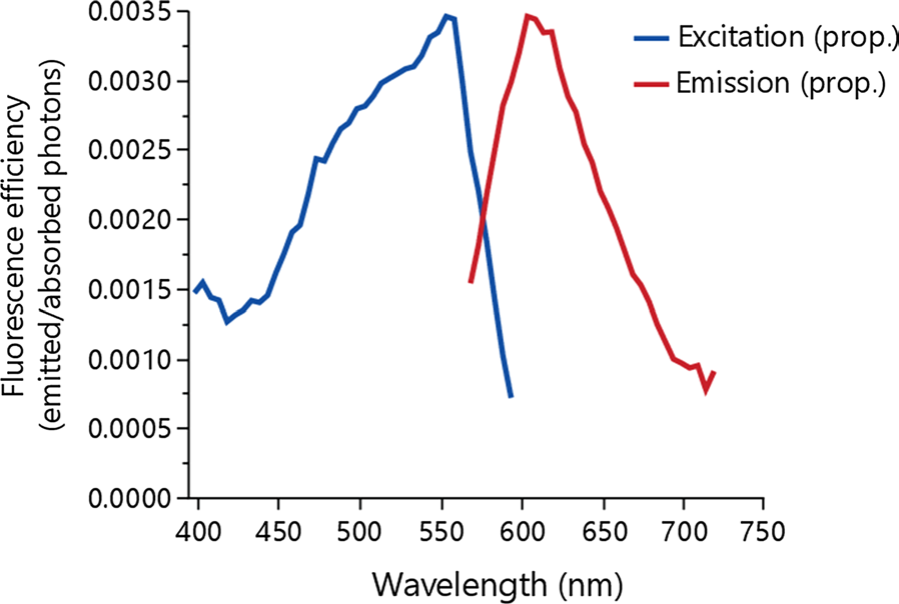
Excitation versus emission peaks for *Tripterygion delaisi* iris fluorescence

Excitation and emission of iris fluorescence is shown in Fig. 3 with excitation being highest at 550 nm and fluorescence emission peaking at 600 nm [26]. Since *T. delaisi* forages at relatively small distances to prey of a few centimeters only, absorption and scattering is negligible (<1% at 600–650 nm at 4 cm distance, [16, 17]). To calculate the decrease of fluorescence brightness over distance, we fixed an eye of *T. delaisi* on a black stick and illuminated it with a blue Hartenberger Mini Compact LCD divetorch (7 × 3.5 W 450 nm bulbs) from a distance of 24 cm. Two short pass filters (Thorlabs FD2C subtractive dichroic color short-pass) were attached in front of the torch to cut out longer wavelengths. Since the eyes quickly darken after decapitating a fish due to the dispersal of melanosomes, we treated the eyes with potassium chloride solution [24] for 1 h to reverse this process before using it. The eye was then oriented downwards at an angle of approximately 45° looking at a diffuse white standard (PTFE). A ruler was placed in line with the outer edge of the iris to serve as a reference. Consecutive measurements were taken in 0.5 cm steps using a calibrated PR 740 SpectraScan Spectroradiometer (Photo Research Inc.,) pointed at the diffuse white standard and measuring reflected fluorescence starting at 0–2.5 cm distance from the eye. The spectrometer was set to 2 nm bandwidth, an aperture of 0.5, with smart dark enabled at a normal speed with an extended exposure time, and was operated with a calibrated MS-75 lens.

**Fig. 4:**
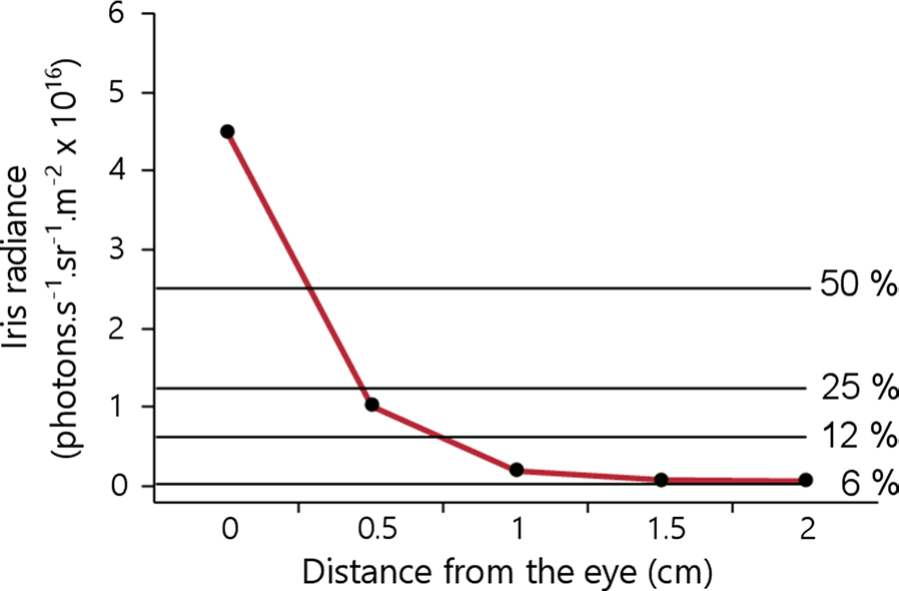
Mean iris fluorescence decline with distance. Percentages indicate the proportion of light *left* at a given distance

The spectrometer used to take measurements adjusts gain to avoid saturation in the brightest wavelengths resulting in noisy measurements in the longer wavelength range. We therefore used an orange filter (Lee filters, Double C.T. Orange 287) attached in front of the spectrometer lens to suppress the excitation light. Radiance measurements were then corrected for the transmission of the used filter and converted to photon radiance by multiplying the measurements with wavelength*5.05*10^15^ [30]. Photon radiance was then summarized between 600 and 650 nm and averaged among the two measured eyes. Figure 4 displays the exponential loss of iris fluorescence with distance. Note that measurements were taken from a diffuse white standard reflecting all wavelengths equally in a 180° angle. These measurements are therefore very conservative compared to a reflector such as a copepod nauplius eye.

For easier comprehensibility, we provide a demonstration of how red fluorescence behaves with increasing/decreasing proportion of longer wavelengths present in the light environment. Since *T. delaisi* is able to quickly regulate its fluorescence we did not conduct this demonstration with live triplefins but used a special mixture of fluorescent paint which resembles different intensities of fluorescence emission of *T. delaisi* (Additional file 5). We then illuminated the fluorescent patches along with a non-fluorescent red diffuse reflectance standard (Labsphere SCS-RD-010) from a distance of 24 cm with the euryspectral and stenospectral light treatment used during the second experiment. The demonstration shows that with decreasing proportion within the longer wavelength range fluorescence appears more intense while in direct comparison the non-fluorescent red standard becomes grey.

### Statistical analysis of fish behaviour

Data were analysed using a generalized linear mixed model using the lme4 package [31] of R [32]. The response variable *n copepods caught*, was modelled as a binomial (*n copepods caught*/*n copepods missed*) response variable with logit link. In both experiments all initial models contained *light treatment*, *bench*, and *week* as well as all biologically relevant interactions as fixed components. To account for the repeated measurements per fish, fish ID was integrated as a random factor with random slopes. An observation-level random factor (random effect that models extra-Poisson variation of count data, [33]) was added as well to account for overdispersion. By using the Bayesian information criterion (BIC), a backward model selection was performed on random (excluding fish ID) and fixed factors to identify the best fitting model with the fewest predictors. Here, we only present the final models including proxies for the goodness-of-fit of the complete model (conditional *R* ^2^) as well as the fixed component (marginal *R* ^2^) [34]. Proxies were calculated using the pairwise SEM package for R [35]. Wald *z* tests were performed to assess the overall significance of fixed effects. All other statistical tests as given, two-way ANOVAs and paired *t* tests were carried out using JMP 11 (SAS) after confirming normality and homoscedasticity.

All data necessary to reproduce our conclusions are provided in the supplementary files section (Additional files 6, 7).

### Pre-results: participation and exclusion criterion

In the first experiment, 31 out of 40 fish participated throughout the entire study whereas the remaining 9 showed no interest and were therefore excluded from the analysis. In the second experiment, 37 of 39 fish successfully participated throughout the whole duration of the experiment. However, 3 males changed to male breeding coloration during the experiment and were excluded from further analysis. Males in breeding coloration increase the content of melanophores in the iris which reduces expressed red fluorescence (unpubl. data). We therefore only considered adults in our analyses that showed their cryptic coloration throughout the entire study.

### Pre-results: spectral treatments

In the second experiment there was no detectable difference between the two stenospectral treatments, that differed only slightly in the spectral range (blue-green), which is why these data were pooled together (paired *t* test: *dF* = 33 *t* = 0.25, *p* = 0.81).

### Pre-results: control treatments

Out of 322 recorded control videos (no copepods), only 12 feeding strikes were observed, eleven of which occurred during the second run where fish had previously received copepods. It is therefore likely that these fish caught copepods that were still around from the earlier runs on that day.

## Results

### Experiment 1: feeding success under shaded conditions

**Fig. 5:**
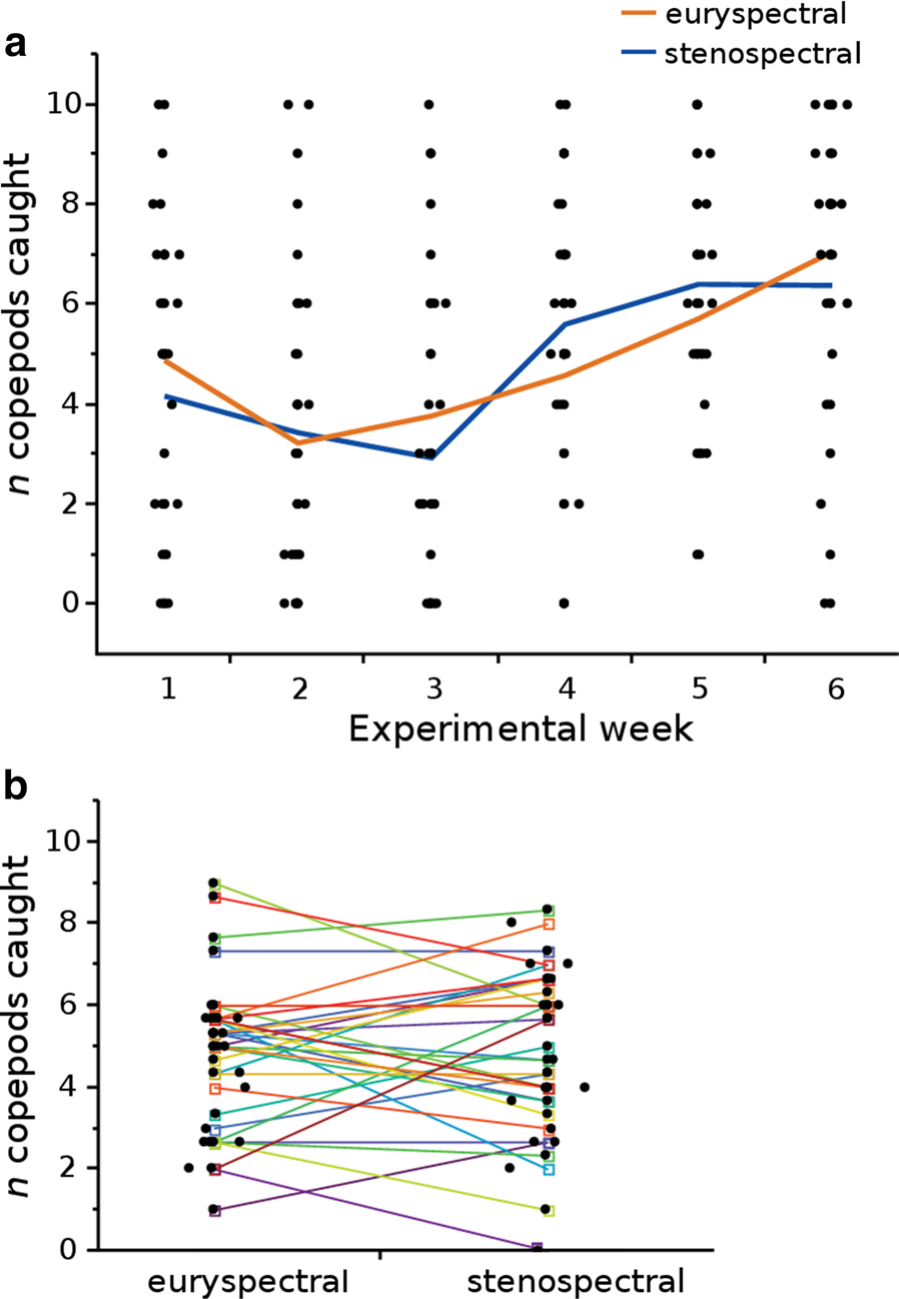
**a** Total number of copepods caught during the first (*shaded*) experiment (*n* = 31). *Black dots* represent the total number of copepods caught by individuals per experimental week in the euryspectral or stenospectral light treatment. *Lines* represent mean copepods caught per week in the respective light treatment. **b** Mean copepods caught per fish and spectral treatment. Lines connect mean values for copepods caught of each individual between the two spectral treatments

There was no difference in the number of copepods caught under euryspectral versus stenospectral conditions under shaded illumination (Fig. 5; Table 1). Fish caught on average 4.83 ± 1.98 SD copepods in the euryspectral treatment and 4.88 ± 1.82 SD in the stenospectral treatment. Similarly, the time it took until 5 out of 10 copepods were caught (“copepod half-time”), did also not differ between light treatments (Additional file 8).

**Table 1:** Foraging success and copepod half-time in response to light treatments

Experiment	Parameter	Std-beta coefficient estimate	SE	z	p	Conditional R^2^	Marginal R^2^
1: Shaded conditions: foraging success	Intercept	−1.323	0.29	−4.47	<0.001		
	Week	0.34	0.06	5.49	<0.001	0.157	0.058
	Light treatment	0.02	0.21	0.14	0.89		
2: Heavily shaded conditions: foraging success	Intercept	0.73	0.15	5.01	<0.001		
	Light treatment	−0.34	0.17	−1.98	0.047	0.063	0.007

Interestingly, however, fish significantly increased their foraging success irrespective of the light treatment from 4.5 ± 2.9 SD copepods in week 1 to 6.7 ± 2.8 SD in week 6 (Fig. 3; Table 1), indicating a learning effect.

### Experiment 2: foraging success under heavily shaded light conditions

**Fig. 6:**
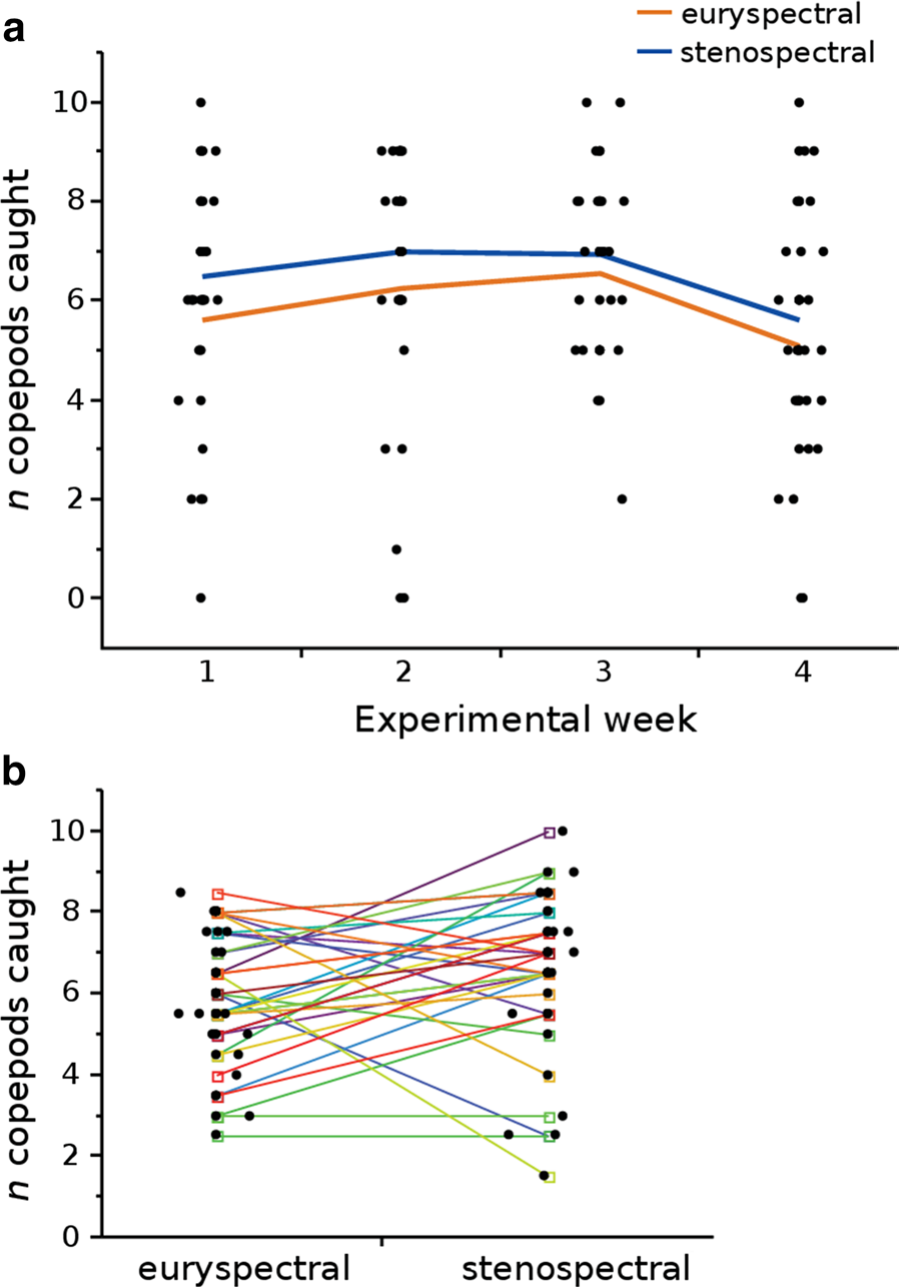
**a** Total number of copepods caught during the second (*heavily shaded*) experiment (*n* = 34). *Black dots* represent the total number of copepods caught by individual per experimental week in the euryspectral or stenospectral light treatment. *Lines* represent mean copepods caught per week in the respective light treatment. **b** Mean copepods caught per fish and spectral treatment. Lines connect mean values for copepods caught of each individual between the two spectral treatments

Fish held in the stenospectral treatment caught on average 6.5 ± 1.97 SD copepods while fish tested under euryspectral conditions only caught 5.8 ± 1.63 SD copepods, a significant difference (Table 1; Fig. 6). Hence, the use of red fluorescence under blue-green illumination in deeper *and* more shaded habitats, allows *T. delaisi* to increase its foraging success by an average of 7%. However, fish showed quite some variation. More than one third (44.4%) of the fish for example, increased their foraging success by 15% on average and 29% of all fish increased their efficiency by at least 20% under stenospectral conditions relative to euryspectral conditions. The highest mean increase for any individual was twofold (9 copepods caught compared to 4.5). In contrast, 8 out of 34 fish showed a higher foraging success in the euryspectral treatment compared with the stenospectral treatment (7.3 ± 1 SD compared with 4.9 ± 2 SD copepods). Similar to the first (shaded) experiment, copepod half-time was not affected by treatment (Additional file 9). Improved performance over the course of the experiment could not be confirmed (no effect of week, Table 1), but experiment 1 ran for 6 weeks, experiment 2 for 4 weeks.

When comparing both experiments (shaded and heavily shaded), fish were generally more successful at catching copepods under heavily shaded conditions (average shaded experiment: 4.86 ± 1.68 SD; heavily shaded experiment: 6.29 ± 1.48 SD). This difference, however, needs to be interpreted with care since the two experiments did not run in parallel, but in successive years, with 2 different fish cohorts.

## Discussion

*Tripterygion delaisi* showed on average a 7% higher foraging success under heavily shaded, blue-green light favoring fluorescence compared with broad-spectral or shaded conditions. Although these results do not represent direct evidence that fish use red fluorescence to enhance their prey detection under stenospectral conditions, they are nevertheless consistent with the active photolocation hypothesis. Assuming that red fluorescent irides indeed facilitate prey detection, they probably do so under heavily shaded conditions only, either under rocks or overhangs where the light is dominated by side-welling blue-green scatter from the open water, or at depths, times of day or degrees of cloud cover, where bright and broad-spectral light is lacking.

Hence, when fish are hunting in sunlit sites, fluorescence is likely to be of little help for foraging. However, it coincides well with *T. delaisi*’s preference for rocky substrates with crevices and overhangs where brightness transitions are frequent and strong, regardless of depth. Under these conditions, red fluorescence might offer a significant advantage when foraging in the shade. Similar benefits would exist when foraging during dusk and dawn.

### Predator–prey interaction

While fish could have theoretically generated a contrast between red illuminated prey and background, explaining the observed increase in foraging success, they could have also used their red fluorescent irides to attract prey. Similar to phototaxis in diurnal vertical migrating invertebrates [36, 37], *T. californicus* could be attracted by certain light cues. This, however, would require copepods to be sensitive to longer wavelengths. Although studies on visual sensitivity of *T. californicus* are rare (but see [20]), our own results obtained from the first pre-experimental study on light preferences of *T. californicus* suggest that such differentiation is absent for the light conditions used here (Additional file 1, red light treatment). However, longer wavelengths are quickly absorbed, implying that red fluorescence can only be effective over very short distances. This is compatible with the saltatory foraging and short-distance strikes typical for *T. delaisi* [27, 28]. Over such short distances, fluorescence could be strong enough to create the proposed contrast. A recent study by Anthes et al. [10] strengthens this hypothesis by showing that red fluorescent irides are a common feature among small benthic predatory fish that predominantly hunt for small invertebrates.

Additionally, wavelengths above 570 nm are rapidly absorbed over larger distances [16, 17, 38]. *T. delaisi* could therefore use red fluorescence to forage more efficiently while remaining hidden from predators nearby.

### Individual variation

Fish tested under heavily shaded light showed substantial individual differences despite the fact that fish were given sufficient time to adjust. Such differences were also present in a previous study of a phenotypic response to different light environments [12]. We propose that this degree of variability may represent a form of microhabitat specialization in this very cryptic species. Fish predominantly foraging in exposed sites may show weaker fluorescence because it is less functional and its absence prevents attracting (red-sensitive) visual predators. Whereas fish that predominantly forage in the shade face the opposite situation. Individual variability also explains the small size of the effect found in the heavily shaded experiment, despite the very strong effects in some individuals. Future work could specifically compare fish collected from exposed sites versus fish collected from overhangs to confirm this view.

### Does brightness perception influence foraging success?

In order to keep the overall brightness similar in all spectral treatments, we increased the abundance of blue and green wavelengths within the stenospectral treatment. *T. delaisi* shows highest sensitivity in the green wavelength range [26]. The stenospectral treatment might therefore have been perceived brighter by the fish, regardless of the real overall brightness. We attempted to take care of this by including a second even “greener” stenospectral treatment and comparing foraging success between the two stenospectral treatments. Since foraging success did not differ between these two treatments, a difference in brightness perception between stenospectral and euryspectral treatment alone cannot explain the observed increase in foraging success in the second, heavily shaded experiment. Furthermore, if perceived brightness indeed affected foraging success, a similar effect would have been present in the first, brighter experiment. Although no such effect could be found, we cannot entirely exclude that perceived brightness might still have had a small effect on foraging success in *T. delaisi*.

## Conclusions

Summarizing, this study shows that *T. delaisi* forages more efficiently under heavily shaded, blue-green light conditions compared with broad light. Assuming that fish may be using red fluorescent emission to enhance prey detection, this result suggests that the functionality of such a mechanism is more plausible over short distances, under stenospectral, shaded conditions. This offers important clues for the design of future experiments to test active photolocation in this system.

## Additional files

**Additional file 1.** Pilot study: behavior of *Tigriopus californicus* under two different spectra.

**Additional file 2.** Distraction pattern on polypropylene foil used to cover the walls of the aquaria.

**Additional file 3.** Video sequence of typical foraging behavior in *T. delaisi* recorded during the experiment.

**Additional file 4.** Pilot study: feeding strikes in *Tripterygion delaisi.*

**Additional file 5.** Animated gif illustrating the contrast generated by red fluorescence under the two spectral treatments.

**Additional file 6.** Foraging success data of the first (shaded) experiment.

**Additional file 7.** Foraging success data of the second (heavily shaded) experiment.

**Additional file 8.** R script used to analyze foraging success in the first (shaded) experiment.

**Additional file 9.** R script used to analyze foraging success in the second (heavily shaded) experiment.
