# Supplementary material for: Fish with red fluorescent eyes forage more efficiently under dim, blue-green light conditions
Source: BMC Ecol. 2017 Apr 20;17:18. doi: 10.1186/s12898-017-0127-y (PMC5397785; doi:10.1186/s12898-017-0127-y)
Supplement: Supplementary file 1 — Additional file 1. Pilot study: behavior of Tigriopus californicus under two different spectra. [file 12898_2017_127_MOESM1_ESM.docx]

**Behavior of *Tigriopus californicus* under two different spectra**

Differences in feeding strikes in *T. delaisi* due to spectral treatment may be caused by a different behavior of the live copepods used as prey. Hence, to assess whether *Tigriopus californicus* reacts differently to the two spectral treatments used in the experiments we conducted a pilot study to assess to what extent their movement changed between the euryspectral and stenospectral conditions. To this end, 18 transparent 4 ml cuvettes filled with seawater containing 10-15 copepods were placed on a dark underground in front of a black background, illuminated with an infrared light source (Versiton SAL-30 IR Illuminator 77 LEDS 30M (100') 12VDC 1.5A, peak at 844 nm). Swimming behavior was then recorded using an infrared sensitive camera (uEye UI-1440 M/C USB2.0) with a macro-lens (MLH-10X, 1/2" 13-130 mm Computar) and an IR pass-filter attached to the lens. This setup visualized copepods as white moving dots on a dark background. The position of the cuvette as well as the distance between the cuvette and the camera was 21 cm. The copepods received the same two spectral treatments used in experiment one and an additional red-light-only treatment (red channel = 100 %, all other channels = 0 %) in a randomized order. Before a treatment started, the lights were turned off for one minute to allow the copepods to calm down. Afterwards, the first light treatment started and the copepods were given one minute to adapt to the new light conditions. The swimming behavior was recorded for 2 min, followed by another 1 min darkness and the next light treatment. The same procedure was carried out for the spectral treatments used in the second experiment (*n* = 18 cuvettes). Videos were analyzed using Image J 1.47v with a self-written macro plugin. All videos were transformed into black and white picture stacks in which the movements of the copepods were visualized as white spots. The picture stacks were then summarized leading to a final picture were the cumulative presence of all copepods in the cuvette could be expressed as a raw intensity value per pixel. The cuvette area in the picture was then divided into an upper and lower part. Using the proportion of copepod presence between the two produced a ratio that indicated in which half the copepods frequented more during the different light treatments.

**Results and conclusion**

Statistical analyses of copepod movement were carried out using JMP 11 (SAS). The proportions of copepod presence were transformed using a square-root ArcSin correction. A nested two-way ANOVA was performed using the corrected *proportion* as response variable, the *spectral treatment* and the *order of treatments* as fixed factors and the *cuvette ID* as random factor. The two-way ANOVA showed that neither the order in which the treatments were presented (two-way ANOVA _bright experiment_: *F* = 2.02, *dF* = 5, *p* = 0.12; two-way ANOVA _dark experiment_: *F* = 2.65, *dF* = 5, *p* = 0.08) nor the spectral treatments had a significant effect on the time the copepods spent in one of the two half´s of the cuvette (two-way ANOVA _bright experiment_: *F* = 0.0005, *dF* = 2, *p* = 0.99; two-way ANOVA _dark experiment_: *F* = 1.6, *dF* = 2, *p* = 0.21). We conclude that *Tigriopus californicus* does not respond behaviorally to the difference between the two light treatments. It therefore seems unlikely that this may explain the treatment effect in the feeding strikes of *T. delaisi.*
