# Supplementary material for: Fish with red fluorescent eyes forage more efficiently under dim, blue-green light conditions
Source: BMC Ecol. 2017 Apr 20;17:18. doi: 10.1186/s12898-017-0127-y (PMC5397785; doi:10.1186/s12898-017-0127-y)
Supplement: Supplementary file 2 — Additional file 2. Distraction pattern on polypropylene foil used to cover the walls of the aquaria. [file 12898_2017_127_MOESM2_ESM.pdf]

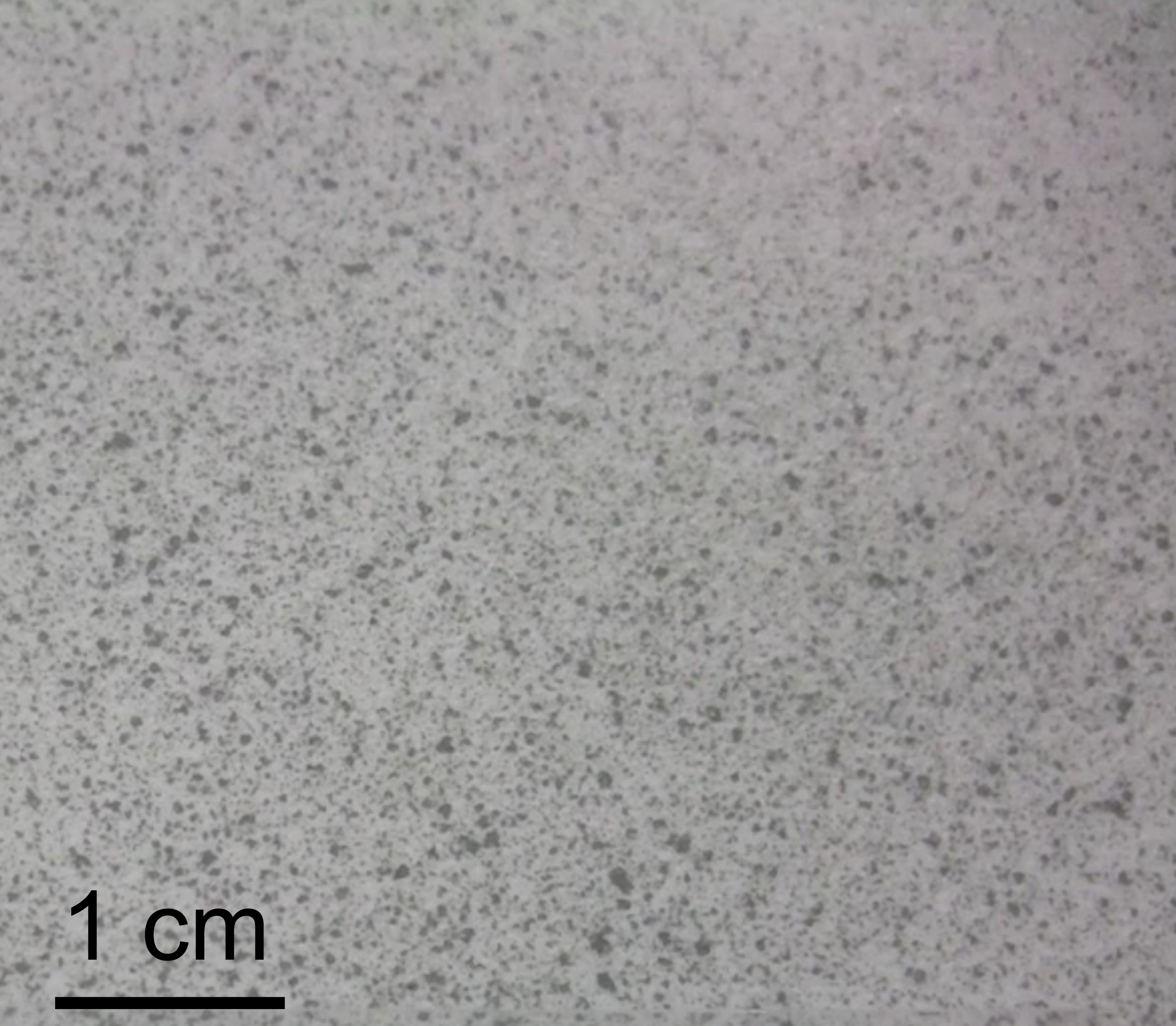
1 cm
