# Supplementary material for: Fish with red fluorescent eyes forage more efficiently under dim, blue-green light conditions
Source: BMC Ecol. 2017 Apr 20;17:18. doi: 10.1186/s12898-017-0127-y (PMC5397785; doi:10.1186/s12898-017-0127-y)
Supplement: Supplementary file 4 — Additional file 4. Pilot study: feeding strikes in Tripterygion delaisi. [file 12898_2017_127_MOESM4_ESM.docx]

**Feeding strikes in *Tripterygion delaisi***

**Goal and methodology**

Does *T. delaisi* only strike at something when it sees prey? In a pilot study we tested how strongly picking behavior by *T. delaisi* is linked to copepod presence. After familiarizing fish with pipette tips (see section fish habituation and testing – methods section main text) 15 fish received six different copepod treatments in which either 1, 2, 3, 4, 6 or 10 live *T. californicus* copepods were administered to their tank in 1 ml salt water. All fish received only one treatment per day in a randomized order across 6 different days to ensure that no copepods were left in the aquarium from prior trials. The picking behavior was then directly observed by the experimenter for a total of 10 min. Since copepods were prepared in advance, stored in 1 ml Eppendorfer tubes, and labeled with the fish ID only, the observer was blind to the actual number of copepods added to the aquarium.

**Results and conclusions**

Out of 15 fish only 12 participated. The remaining three individuals were excluded from analysis. The results show that the number of picking could be very precisely explained by the number of copepods administered (Figure). We never saw more picks than the number of copepods provided. Due to the repeated measures design we only used the mean number of *copepods caught between the individuals per treatment (n copepods administered)* to calculate the linear regression: *R^2^* = 0.94, *df_effec_t* = 1, df_error_= 4 *F* = 74.78, *p* = 0.001). We conclude that feeding strikes are indeed a good measure of feeding success in *T. delaisi*.

Figure: Relationship between number of copepods administered and the number of feeding strikes of *Tripterygion delaisi* (*n* = 12).
