# Supplementary figures and images for: Fish with red fluorescent eyes forage more efficiently under dim, blue-green light conditions
Source: BMC Ecol. 2017 Apr 20;17:18. doi: 10.1186/s12898-017-0127-y (PMC5397785; doi:10.1186/s12898-017-0127-y)

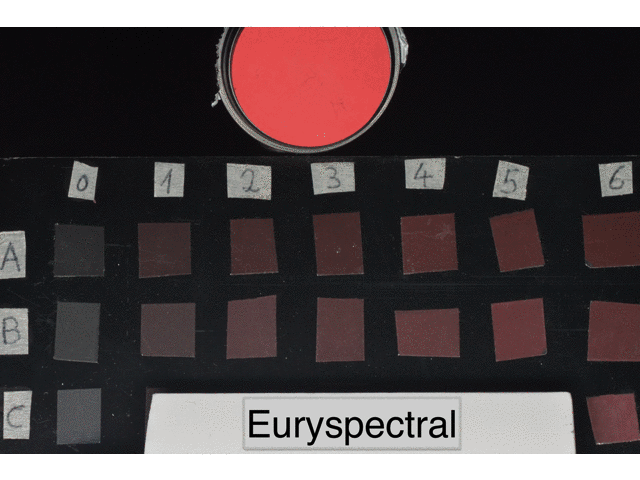

Supplement: Supplementary file 5 — Additional file 5. Animated gif illustrating the contrast generated by red fluorescence under the two spectral treatments. [file 12898_2017_127_MOESM5_ESM.gif]
