# Supplementary material for: Fish with red fluorescent eyes forage more efficiently under dim, blue-green light conditions
Source: BMC Ecol. 2017 Apr 20;17:18. doi: 10.1186/s12898-017-0127-y (PMC5397785; doi:10.1186/s12898-017-0127-y)
Supplement: Supplementary file 8 — Additional file 8. R script used to analyze foraging success in the first (shaded) experiment. [file 12898_2017_127_MOESM8_ESM.pdf]

```
R version 3.2.3 (2015-12-10)

> #####################################################
> # Foraging analysis in Triplefins; bright experiment, Ulrike Harant
> #####################################################

> rm(list=ls()) #remove all previous info from workspace

> # Set paths at Uni

> setwd("C:/ulli/FLUO_UH/statistic/ULLIS_STICK/nils")
> library(lme4)

Lade nötiges Paket: Matrix
Warning message:
Paket ‘lme4’ wurde unter R Version 3.2.5 erstellt

> library(pastecs)  # for descriptive stats

> # ***************************************************************
> # Function to test for overdispersion in any of the used models #

> dispersion_glmer <- function(modelglmer)
+ {n <- length(resid(modelglmer))
+ return(  sqrt( sum(c(resid(modelglmer),modelglmer@u) ^2) / n ) )
+ }

> # call function as follows:
> #dispersion_glmer(Modelname) #should be between, 0.75 and 1.4 if not under-
or overdispersed, respectively

> # ***************************************************************
> #################
> # Load data files
> #################

> dat<-read.table("2015.02.20_resultsR.csv", header=T, sep=",")

> # some new variables used during the analyses

> dat$fishID <- as.factor(dat$fishID)
> dat$obsid<-factor(1:nrow(dat)) # create column that identifies each individu
al observation

> #  ANALYSIS A: copepod retention time ****
> #**********************************
> #**********************************
> #----------------------------------------------------------------------------
-----------------#

> # Step 1: Optimze random component (while keeping all RELEVANT fixed effects
+ interactions).
> #----------------------------------------------------------------------------
-----------------#
> # First we check for overdispersion in the most complete model, i.e. compare
dispersion parameters and model fit among the full model and a model that incl
udes obsid()
```

```
> mod1.full<-glmer(c5 ~
+ week + lighttreatment + roomside +
+ (lighttreatment|fishID)+ (1|obsid), family=poisson, data=dat) #

> #with random slopes + random intercept per individual across Backgrounds and
across Copepods

> mod1.a<- update(mod1.full, .~. -(1|obsid))

> dispersion_glmer(mod1.full) #near 1 as required

[1] 1.000001

> dispersion_glmer(mod1.a) # near 2, indicating  overdispersion, so leave obsi
d in
[1] 136.3378

> anova(mod1.full,mod1.a) # the model including obsid is clearly better in ter
ms of model fit, so we continue using this one.

Data: dat
Models:
mod1.a: c5 ~ week + lighttreatment + roomside + (lighttreatment | fishID)
mod1.full: c5 ~ week + lighttreatment + roomside + (lighttreatment | fishID) +
mod1.full:     (1 | obsid)
          Df     AIC     BIC   logLik deviance   Chisq Chi Df Pr(>Chisq)
mod1.a     7 2307236 2307256 -1153611  2307222
mod1.full  8    3098    3121    -1541     3082 2304140      1  < 2.2e-16 ***
---
Signif. codes:  0 ‘***’ 0.001 ‘**’ 0.01 ‘*’ 0.05 ‘.’ 0.1 ‘ ’ 1

> # Second, check the relevance of looking for random slopes over lighttreatme
nts

> mod1.a<-update(mod1.full, .~. -(lighttreatment|fishID) + (1|fishID))  # now
only random intercept per fishID

> anova(mod1.full,mod1.a) #AICs, BICs, and LR-tests, stepwise

Data: dat
Models:
mod1.a: c5 ~ week + lighttreatment + roomside + (1 | obsid) + (1 | fishID)
mod1.full: c5 ~ week + lighttreatment + roomside + (lighttreatment | fishID) +
mod1.full:     (1 | obsid)
          Df    AIC    BIC  logLik deviance  Chisq Chi Df Pr(>Chisq)
mod1.a     6 3094.5 3111.4 -1541.3   3082.5
mod1.full  8 3098.3 3120.8 -1541.1   3082.3 0.2359      2     0.8887

> # All 3 parameteres agree: random slopes not necessary, so drop

> mod2.full<- mod1.a

> #-----------------------------------------------------------------------------#
> # Step 3: We check the FIXED component by sequential model comparisons.
> #-----------------------------------------------------------------------------#
> ### Round 1 of model selection: Deletion of the highest order main effects #
##

> mod3.a <- update(mod2.full, .~. - week)
```

```
> mod3.b <- update(mod2.full, .~. - roomside)

> # the following lines compare each of our simplified models against our curr
ent full model
> anova(mod3.a,mod2.full)

Data: dat
Models:
mod3.a: c5 ~ lighttreatment + roomside + (1 | obsid) + (1 | fishID)
mod2.full: c5 ~ week + lighttreatment + roomside + (1 | obsid) + (1 | fishID)
          Df    AIC    BIC  logLik deviance  Chisq Chi Df Pr(>Chisq)
mod3.a     5 3112.2 3126.2 -1551.1   3102.2
mod2.full  6 3094.5 3111.4 -1541.3   3082.5 19.629      1  9.403e-06 ***
---
Signif. codes:  0 ‘***’ 0.001 ‘**’ 0.01 ‘*’ 0.05 ‘.’ 0.1 ‘ ’ 1
> anova(mod3.b,mod2.full)
Data: dat
Models:
mod3.b: c5 ~ week + lighttreatment + (1 | obsid) + (1 | fishID)
mod2.full: c5 ~ week + lighttreatment + roomside + (1 | obsid) + (1 | fishID)
          Df    AIC    BIC  logLik deviance  Chisq Chi Df Pr(>Chisq)
mod3.b     5 3094.5 3108.6 -1542.2   3084.5
mod2.full  6 3094.5 3111.4 -1541.3   3082.5 1.9426      1     0.1634

> # Roomside = mod3.b has clearly the least significant contribution and lowes
t BIC, so this is the first fixed effect to drop!!
> ### Round 2 of model selection ###
> # Our new full model is

> mod3.full <- mod3.b

> mod4.a <- update(mod3.full, .~. - week)

> anova(mod4.a,mod3.full)

Data: dat
Models:
mod4.a: c5 ~ lighttreatment + (1 | obsid) + (1 | fishID)
mod3.full: c5 ~ week + lighttreatment + (1 | obsid) + (1 | fishID)
          Df    AIC    BIC  logLik deviance  Chisq Chi Df Pr(>Chisq)
mod4.a     4 3111.8 3123.1 -1551.9   3103.8
mod3.full  5 3094.5 3108.6 -1542.2   3084.5 19.311      1   1.11e-05 ***
---
Signif. codes:  0 ‘***’ 0.001 ‘**’ 0.01 ‘*’ 0.05 ‘.’ 0.1 ‘ ’ 1

> # Week remains significant, is therefore maintained in the model

> Mod_final <- mod3.full

> Mod_final<-glmer(c5 ~
+ week + lighttreatment+
+ (1|fishID)+ (1|obsid), family=poisson, data=dat)

> summary(Mod_final)

Generalized linear mixed model fit by maximum likelihood (Laplace  Approximati
on)
 [glmerMod]
```

```
 Family: poisson  ( log )
Formula: c5 ~ week + lighttreatment + (1 | fishID) + (1 | obsid)
   Data: dat

     AIC      BIC   logLik deviance df.resid 
  3094.5   3108.6  -1542.2   3084.5      119 

Scaled residuals: 
       Min         1Q     Median         3Q        Max 
-0.0314583 -0.0045540  0.0007275  0.0021501  0.0069223 

Random effects:
 Groups Name        Variance Std.Dev.
 obsid  (Intercept) 0.6268   0.7917  
 fishID (Intercept) 0.1620   0.4026  
Number of obs: 124, groups:  obsid, 124; fishID, 34

Fixed effects:
                      Estimate Std. Error z value Pr(>|z|)    
(Intercept)           12.02616    0.20411   58.92  < 2e-16 ***
week                  -0.19204    0.04111   -4.67 2.99e-06 ***
lighttreatmentshallow -0.20186    0.14534   -1.39    0.165    
---
Signif. codes:  0 ‘***’ 0.001 ‘**’ 0.01 ‘*’ 0.05 ‘.’ 0.1 ‘ ’ 1

Correlation of Fixed Effects:
            (Intr) week 
week        -0.795      
lghttrtmnts -0.361  0.015

> # Effect tests: Likelihood ratio tests

> Mod_lighttreat_effect <- update(Mod_final, .~. - lighttreatment)

> anova(Mod_final,Mod_lighttreat_effect)

Data: dat
Models:
Mod_lighttreat_effect: c5 ~ week + (1 | fishID) + (1 | obsid)
Mod_final: c5 ~ week + lighttreatment + (1 | fishID) + (1 | obsid)
                      Df    AIC    BIC  logLik deviance Chisq Chi Df Pr(>Chisq
)
Mod_lighttreat_effect  4 3094.4 3105.7 -1543.2   3086.4                        
Mod_final              5 3094.5 3108.6 -1542.2   3084.5 1.919      1      0.16
6
```
