# Supplementary material for: Fish with red fluorescent eyes forage more efficiently under dim, blue-green light conditions
Source: BMC Ecol. 2017 Apr 20;17:18. doi: 10.1186/s12898-017-0127-y (PMC5397785; doi:10.1186/s12898-017-0127-y)
Supplement: Supplementary file 9 — Additional file 9. R script used to analyze foraging success in the second (heavily shaded) experiment. [file 12898_2017_127_MOESM9_ESM.pdf]

```
R version 3.2.3 (2015-12-10) -- "Wooden Christmas-Tree"

#####################################################
# Foraging analysis in Triplefins dim experiment, Ulrike Harant
#####################################################

rm(list=ls()) #remove all previous info from workspace

# Set paths at Uni

> setwd("C:/ulli/FLUO_UH/foraging experiment 2016")
> library(ggplot2)
> library(lme4)

Lade nötiges Paket: Matrix
Warning message:
Paket ‘lme4’ wurde unter R Version 3.2.5 erstellt

> dispersion_glmer <- function(modelglmer)
+ {n <- length(resid(modelglmer))
+ return(  sqrt( sum(c(resid(modelglmer),modelglmer@u) ^2) / n ) )
+ }

> dat<-read.table("foragingsuccess2016.txt", header=T, sep=",")
> dat$fishID <- as.factor(dat$fishID)
> dat$obsid<-factor(1:nrow(dat)) # create column that identifies each individu
al observation

> mod1.full<-glmer(C5 ~
+ week + lighttreatment + roomside +
+ (lighttreatment|fishID)+ (1|obsid), family=poisson, data=dat) #

> mod1.a<- update(mod1.full, .~. -(1|obsid))

> dispersion_glmer(mod1.full) #near 1 as required
[1] 0.9999956

> dispersion_glmer(mod1.a) # near 2, indicating  overdispersion, so leave obsi
d in

[1] 127.362

> anova(mod1.full,mod1.a) # the model including obsid is clearly better in ter
ms of model fit, so we continue using this one.

Data: dat
Models:
mod1.a: C5 ~ week + lighttreatment + roomside + (lighttreatment | fishID)
mod1.full: C5 ~ week + lighttreatment + roomside + (lighttreatment | fishID) +
mod1.full:     (1 | obsid)
          Df     AIC     BIC  logLik deviance   Chisq Chi Df Pr(>Chisq)
mod1.a     7 1900111 1900131 -950049  1900097
mod1.full  8    2872    2894   -1428     2856 1897241      1  < 2.2e-16 ***
---
Signif. codes:  0 ‘***’ 0.001 ‘**’ 0.01 ‘*’ 0.05 ‘.’ 0.1 ‘ ’ 1

> mod1.a<-update(mod1.full, .~. -(lighttreatment|fishID) + (1|fishID))  # now
only random intercept per fishID
```

```
> anova(mod1.full,mod1.a) #AICs, BICs, and LR-tests, stepwise

Data: dat
Models:
mod1.a: C5 ~ week + lighttreatment + roomside + (1 | obsid) + (1 | fishID)
mod1.full: C5 ~ week + lighttreatment + roomside + (lighttreatment | fishID) +
mod1.full:     (1 | obsid)
          Df    AIC    BIC  logLik deviance Chisq Chi Df Pr(>Chisq)
mod1.a     6 2867.8 2884.4 -1427.9   2855.8
mod1.full  8 2871.8 2893.9 -1427.9   2855.8     0      2          1

> mod2.full<- mod1.a

> mod3.a <- update(mod2.full, .~. - week)

> mod3.b <- update(mod2.full, .~. - roomside)

> # the following lines compare each of our simplified models against our curr
ent full model

> anova(mod3.a,mod2.full)

Data: dat
Models:
mod3.a: C5 ~ lighttreatment + roomside + (1 | obsid) + (1 | fishID)
mod2.full: C5 ~ week + lighttreatment + roomside + (1 | obsid) + (1 | fishID)
          Df    AIC    BIC  logLik deviance  Chisq Chi Df Pr(>Chisq)
mod3.a     5 2867.8 2881.6 -1428.9   2857.8
mod2.full  6 2867.8 2884.4 -1427.9   2855.8 1.9582      1     0.1617

> anova(mod3.b,mod2.full)

Data: dat
Models:
mod3.b: C5 ~ week + lighttreatment + (1 | obsid) + (1 | fishID)
mod2.full: C5 ~ week + lighttreatment + roomside + (1 | obsid) + (1 | fishID)
          Df    AIC    BIC  logLik deviance  Chisq Chi Df Pr(>Chisq)
mod3.b     5 2866.4 2880.2 -1428.2   2856.4
mod2.full  6 2867.8 2884.4 -1427.9   2855.8 0.5266      1      0.468

> mod3.full <- mod3.b

> mod4.a <- update(mod3.full, .~. - week)

> anova(mod4.a,mod3.full)

Data: dat
Models:
mod4.a: C5 ~ lighttreatment + (1 | obsid) + (1 | fishID)
mod3.full: C5 ~ week + lighttreatment + (1 | obsid) + (1 | fishID)
          Df    AIC    BIC  logLik deviance Chisq Chi Df Pr(>Chisq)
mod4.a     4 2866.3 2877.3 -1429.2   2858.3
mod3.full  5 2866.4 2880.2 -1428.2   2856.4 1.912      1     0.1667

> # Week can be dropped as well

> mod4.full<- mod4.a

> Mod_final <- mod4.full
```

```
> Mod_final<-glmer(C5 ~ + lighttreatment+ (1|fishID)+ (1|obsid), family=poisso
n, data=dat)

> summary(Mod_final)

Generalized linear mixed model fit by maximum likelihood (Laplace Approximatio
n) ['glmerMod']

 Family: poisson  ( log )
Formula: C5 ~ lighttreatment + (1 | fishID) + (1 | obsid)
   Data: dat

     AIC      BIC   logLik deviance df.resid
  2866.3   2877.3  -1429.1   2858.3      113

Scaled residuals:
       Min         1Q     Median          3Q        Max
-0.0295330 -0.0046178 -0.0009088  0.0038268  0.0069495

Random effects:
 Groups Name        Variance  Std.Dev.
 obsid  (Intercept) 3.439e-01 5.864e-01
 fishID (Intercept) 2.278e-16 1.509e-08
Number of obs: 117, groups:  obsid, 117; fishID, 35

Fixed effects:
                      Estimate Std. Error z value Pr(>|z|)
(Intercept)           11.27017    0.07508  150.10   <2e-16 ***
lighttreatmentshallow  0.12436    0.10853    1.15    0.252
---
Signif. codes:  0 '***' 0.001 '**' 0.01 '*' 0.05 '.' 0.1 ' ' 1

Correlation of Fixed Effects:
            (Intr)
lghttrtmnts -0.692


> # Effect tests: Likelihood ratio tests

> Mod_lighttreat_effect <- update(Mod_final, .~. - lighttreatment)

> anova(Mod_final,Mod_lighttreat_effect)

Data: dat
Models:
Mod_lighttreat_effect: C5 ~ (1 | fishID) + (1 | obsid)
Mod_final: C5 ~ lighttreatment + (1 | fishID) + (1 | obsid)
                      Df    AIC    BIC  logLik deviance  Chisq Chi Df Pr(>Chis
q)
Mod_lighttreat_effect  3 2865.6 2873.9 -1429.8   2859.6
Mod_final              4 2866.3 2877.3 -1429.2   2858.3 1.3056      1     0.25
32

> library(piecewiseSEM)

> sem.model.fits(Mod_final) #provides marginal R² = just fixed effects, and co
nditional R2 = fixed + random component.
     Class  Family Link   Marginal Conditional      AIC
1 glmerMod poisson  log 0.01119306  0.01119306 2866.291
```

```
> # Effect tests: Likelihood ratio tests

> treat_effect <- update(Mod_final, .~. - lighttreatment)

> anova(Mod_final,treat_effect)

Data: dat
Models:
treat_effect: C5 ~ (1 | fishID) + (1 | obsid)
Mod_final: C5 ~ lighttreatment + (1 | fishID) + (1 | obsid)
             Df    AIC    BIC  logLik deviance  Chisq Chi Df Pr(>Chisq)
treat_effect  3 2865.6 2873.9 -1429.8   2859.6
Mod_final     4 2866.3 2877.3 -1429.2   2858.3 1.3056      1     0.2532

> # Effect tests: Chisquare tests using Anova() command

> library(car)

Warning message:
Paket 'car' wurde unter R Version 3.2.5 erstellt

> Anova(Mod_final,type="III")# type III Sum of Squares. Model was fitted using
method=REML

Analysis of Deviance Table (Type III Wald chisquare tests)

Response: C5
                    Chisq Df Pr(>Chisq)
(Intercept)    22530.1897  1     <2e-16 ***
lighttreatment     1.3131  1     0.2518
---
Signif. codes:  0 '***' 0.001 '**' 0.01 '*' 0.05 '.' 0.1 ' ' 1

> install.packages("rmarkdown")
also installing the dependency 'base64enc'
```
